# Tidal amplification and salt intrusion in the Mekong Delta driven by anthropogenic sediment starvation

**DOI:** 10.1038/s41598-019-55018-9

**Published:** 2019-12-10

**Authors:** Sepehr Eslami, Piet Hoekstra, Nam Nguyen Trung, Sameh Ahmed Kantoush, Doan Van Binh, Do Duc Dung, Tho Tran Quang, Maarten van der Vegt

**Affiliations:** 1 Department Physical Geography, Faculty of Geoscience, Utrecht University, Utrecht, 3584 CB the Netherlands; 2 Southern Institute for Water Resources Planning, Ward 2, 72710 Ho Chi Minh City, Vietnam; 3 Disaster Prevention Research Institute, Kyoto University, Uji, 611-0011 Gokasho Kyoto, Japan; 4 Department of Water Resources Engineering, Thuyloi University, 175 Tay Son, Dong Da, Hanoi, Vietnam

**Keywords:** Environmental sciences, Hydrology, Ocean sciences

## Abstract

Natural resources of the Mekong River are essential to livelihood of tens of millions of people. Previous studies highlighted that upstream hydro-infrastructure developments impact flow regime, sediment and nutrient transport, bed and bank stability, fish productivity, biodiversity and biology of the basin. Here, we show that tidal amplification and saline water intrusion in the Mekong Delta develop with alarming paces. While offshore M_2_ tidal amplitude increases by 1.2–2 mm yr^−1^ due to sea level rise, tidal amplitude within the delta is increasing by 2 cm yr^−1^ and salinity in the channels is increasing by 0.2–0.5 PSU yr^−1^. We relate these changes to 2–3 m bed level incisions in response to sediment starvation, caused by reduced upstream sediment supply and downstream sand mining, which seems to be four times more than previous estimates. The observed trends cannot be explained by deeper channels due to relative sea level rise; while climate change poses grave natural hazards in the coming decades, anthropogenic forces drive short-term trends that already outstrip climate change effects. Considering the detrimental trends identified, it is imperative that the Mekong basin governments converge to effective transboundary management of the natural resources, before irreversible damage is made to the Mekong and its population.

## Introduction

The Mekong River (MR) springs from the Tibetan-Qinghai Plateau, runs across six countries and it forms the Vietnamese Mekong Delta (VMD). The lower Mekong basin (LMB) is home to over 70 million people and Mekong riverine resources are indispensable to food and job security of this population^1,2^. In the southern end of LMB, the VMD (4 Mha^3^) accommodates 22% of Vietnam’s population (17 M) and supplies 50% of the nation’s food^4^. The Mekong River Basin (MB), pristine until two decades ago^5^, is now “divided between multiple liberalized economies”^6^ and competition ground over natural resources and disputable infrastructure projects. Consequently, in response to upstream developments and downstream interventions, the VMD experiences river bed^7^, bank^8–10^ and coastal erosion^4^, alarming land subsidence rates^11,12^, flooding^13–18^ and frequently-reported excessive saline water intrusion (SWI)^19,20^. While many studies^21–25^ relate SWI within the VMD to global climate change (CC) and project it as a long-term hazard in response to sea level rise (SLR), other estuarine systems around the world have shown SWI increase in response to anthropogenic morphological changes^26^. By studying a comprehensive dataset, supported by models and observations, this study unveils that anthropogenic channel bed degradation and its corresponding tidal amplification results in increasing SWI trends within the VMD estuarine system.

**Figure 1:**
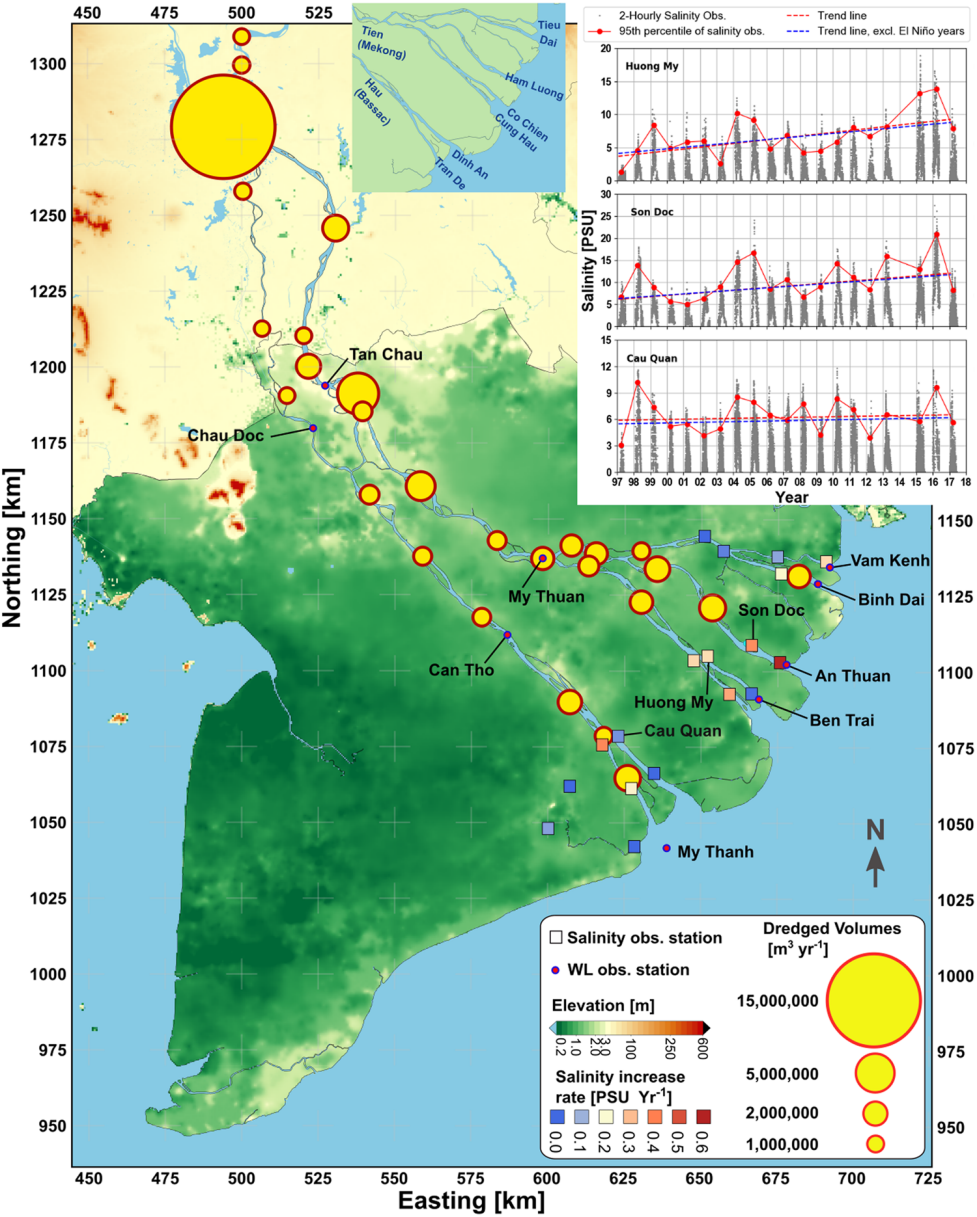
Digital elevation map of the Mekong Delta^30^, including the salinity increase rates at multiple stations, the estimated sand mining volumes (scaled with surface area of the circles); The sand mining figures upstream of the VMD are extracted from previous publications^10^, but updated within the VMD; the top right panel shows three examples of salinity measurement and the P95 trend lines over 20 years; the top panel shows the names of the lower estuarine distributary channels of the Mekong Delta for reference (coord. system WGS84-UTM 48 N).

LMB is characterized by monsoon-dominated seasonal climate with rainfall differentiating between the wet (July-October, SW monsoon) and dry (December-May, NE monsoon) seasons^2^. While Mekong transports 300–550 G m^3^yr^−1^ fresh water^27,28^, monthly average discharge at Kratie can vary between 2–36 10^3^ m^3^ s^−1^ from April to September^29^. Figure 1 shows the VMD elevation^30^, much of which is below 1 m and susceptible to flooding and SWI. During the wet season, SWI is limited to only a few kilometres versus tens of kilometres during the dry season^31^, affecting 1.3 Mha^23,32^. Currently, there are 6 operational mainstream dams along the Lancang-Mekong River^33^, the Xayaburi dam was recently commenced along the mainstream in Laos, and other 170 hydropower and 180 irrigation dams over the Mekong tributaries^34^. Due to hydropower operation, dry season discharge has increased^33,35^, the onset of the wet season is delayed^36^ and the peak of the flood pulse is regulated^33,37^. General consensus is that despite large uncertainties in projecting CC, both seasonal and annual discharge increase under most of the scenarios^18,38–40^. Furthermore, it is expected that the frequency of extreme high flow events increases and the frequency of extreme low flow conditions reduces^40^. Although both hydropower operations and CC lead to increase in freshwater supply and act against dry season SWI, yet, SWI seems to be an every-year concern to the authorities. While various CC adaptation plans are being implemented^22,23^, tidal amplification and SWI are addressed as long term trends attributed to SLR. However, after the 2016 record SWI^20^, following the El Niño of 2015–2016 and frequent reports of high-tide flooding^41–44^, there is an urgent need to introduce another perspective.

### Changes in salinity and tide

**Figure 2:**
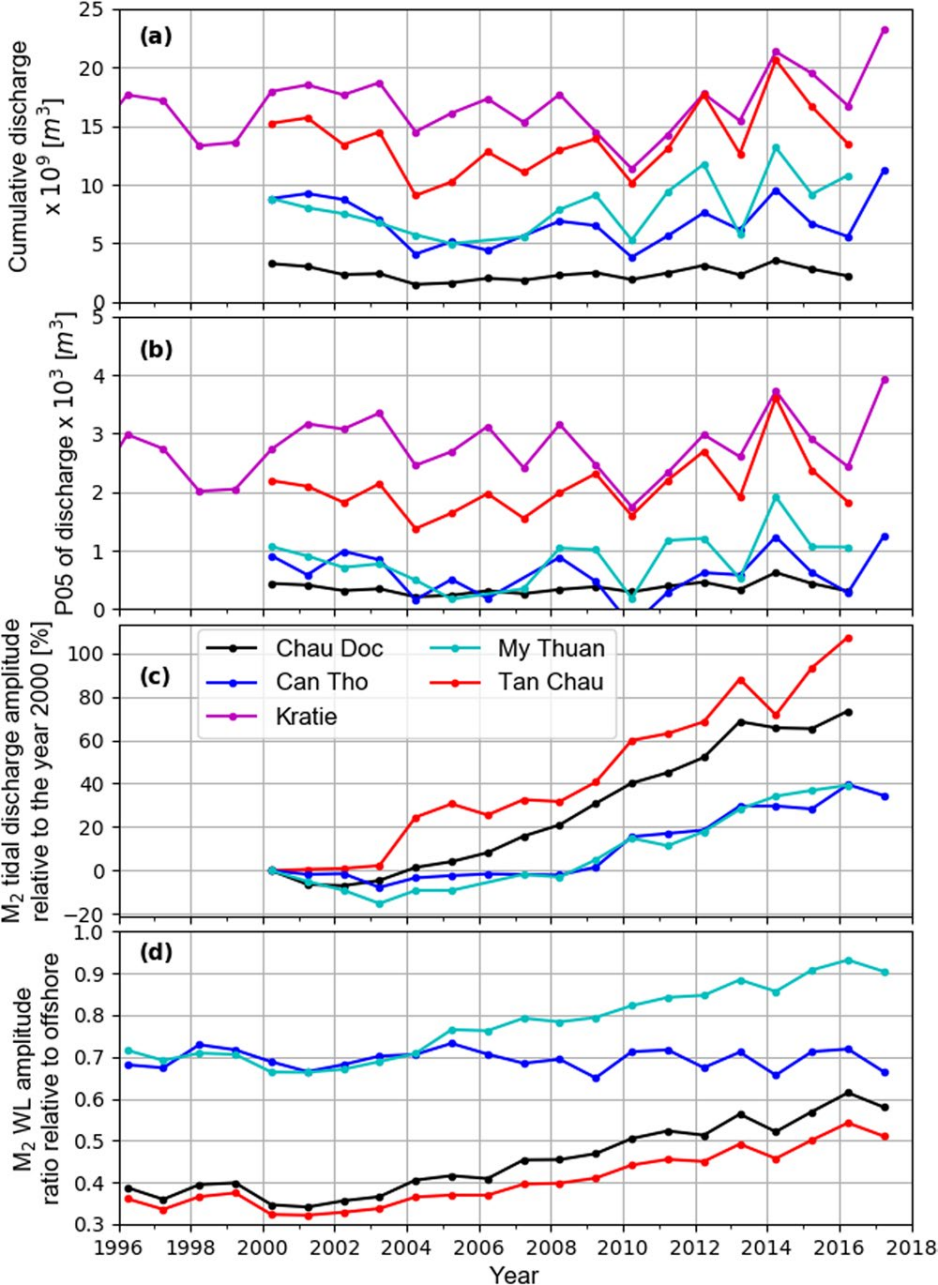
Various tidal and non-tidal hydrodynamic properties of the Mekong Delta during the peak of the dry season (March and April); cumulative discharge at different stations (**a**); 5^th^ percentile (P05) of average discharge (**b**); amplitude of M_2_ tidal discharge relative to the year 2000 (**c**); M_2_ amplitude of tidal water level relative to offshore M_2_ tidal amplitude (**d**).

Mann-Kendall test of the highest 5% (P95) salinity measurements during the dry season shows an average 0.2 PSU yr^−1^ temporal increase over the last two decades at multiple stations (average 50% increase along estuarine channels, in cases up to 100%). The magnitudes of the trends, estimated by Theil-Sen slope (see methods), are shown in Fig. 1. They show a spatial asymmetry in SWI increase; i.e., except Dinh An distributary of Hau River, all channels show temporal increase in SWI. In principle, SWI is the outcome of the competition between fluvial (discharge) and marine (tidal mixing) forces within a given geometry^45–48^. Figure 2a shows that dry season cumulative discharge upstream of the VMD at Kratie, Cambodia, as well as the measurement stations within the VMD, do not show any declining trend in the last two decades. In fact, over a longer period, discharge in Kratie is increasing^18,35,38–40^ (see supplementary information). Furthermore, P05 of discharge, indicating lowest discharge (Fig. 2b), also follows the cumulative discharge trend, suggesting that SWI is increasing despite an increase in freshwater supply to the VMD.

**Figure 3:**
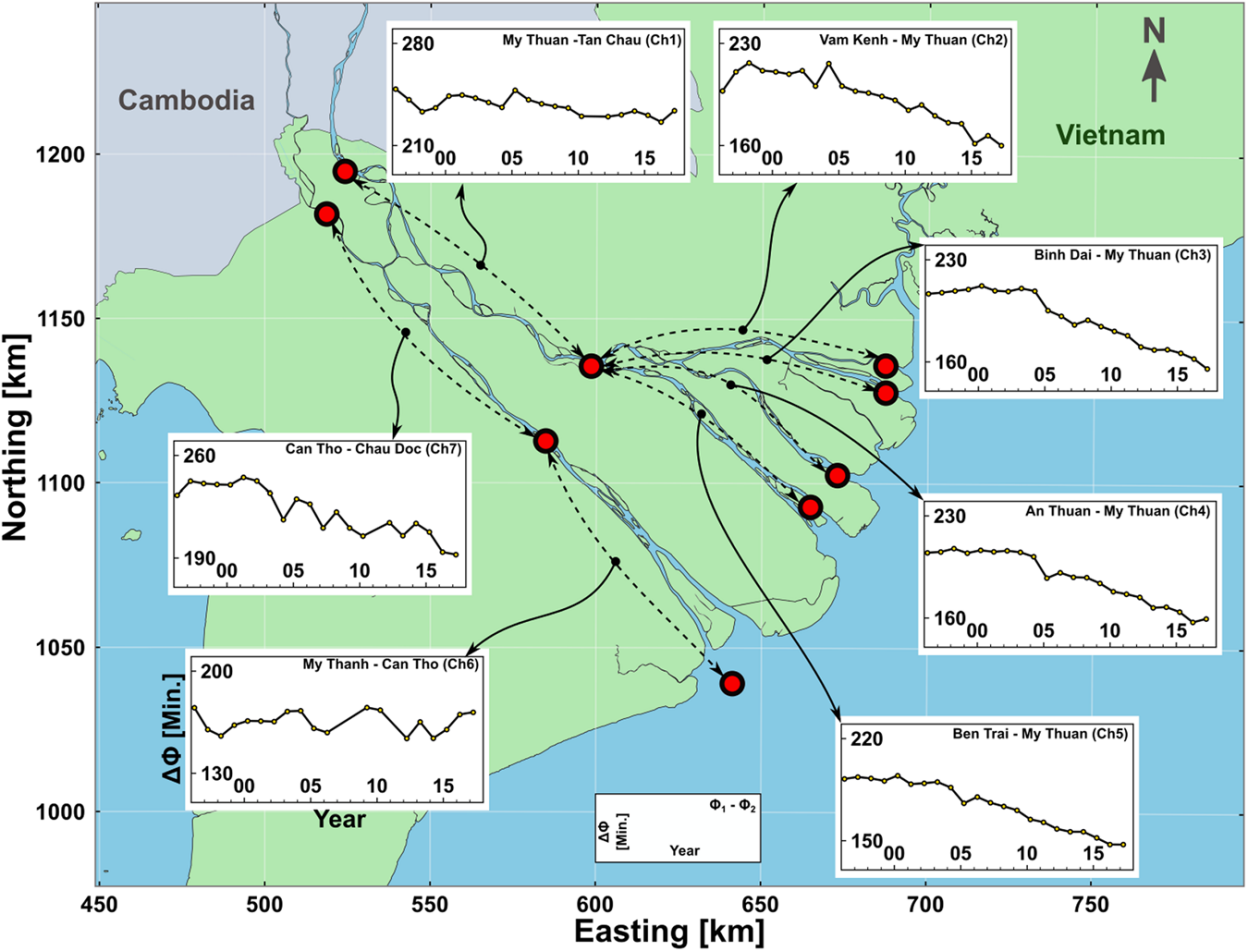
M_2_ phase difference (tidal travel time) between consecutive downstream and upstream stations (coord. system WGS84-UTM 48 N).

The M_2_ tidal discharge amplitude (horizontal tide, Fig. 2c) relative to the year 2000 shows a consistent and significant increasing temporal trend in all stations, varying from 40% in Can Tho and My Thuan with larger tidal discharge, to 100% in Tan Chau with lower tidal discharge. At the same time, M_2_ tidal water level amplitude (vertical tide, Fig. 2d) relative to offshore (see methods, Harmonic Analysis) shows substantial increase, except in Can Tho. While offshore M_2_ tidal amplitude increases by c. 1.2–2 mm yr^−1^ due to SLR^49^, tidal amplification within the VMD, especially within the Tien River, dramatically deviates from that trend and in the past decade, it has increased by c. 1.5–2 cm yr^−1^ (also see supplementary information). This implies that although riverine force (discharge) has increased in time, the strength of the mixing processes has increased significantly due to tidal dynamics within the VMD. In addition, Fig. 3 shows consistent reduction of M_2_ tidal propagation time along different branches of the estuarine system over the past decade (except downstream of Can Tho). The increased tidal propagation speed substantiates attribution of the increased tidal amplitudes to channel incision and rules out impact of other physical properties (e.g., roughness). As the first order shallow water wave celerity scales with $\sqrt{gh}$ ($g$ = gravitational acceleration, *h* = depth), following the linearized asymptotic solution for phase speed in a long, intermediate-depth, near-equilibrium estuary^50^, the reduced phase speed between various stations implies that the channels, on average, have deepened between 2 to 3 m (see also supplementary information).

### Bed level changes

**Figure 4:**
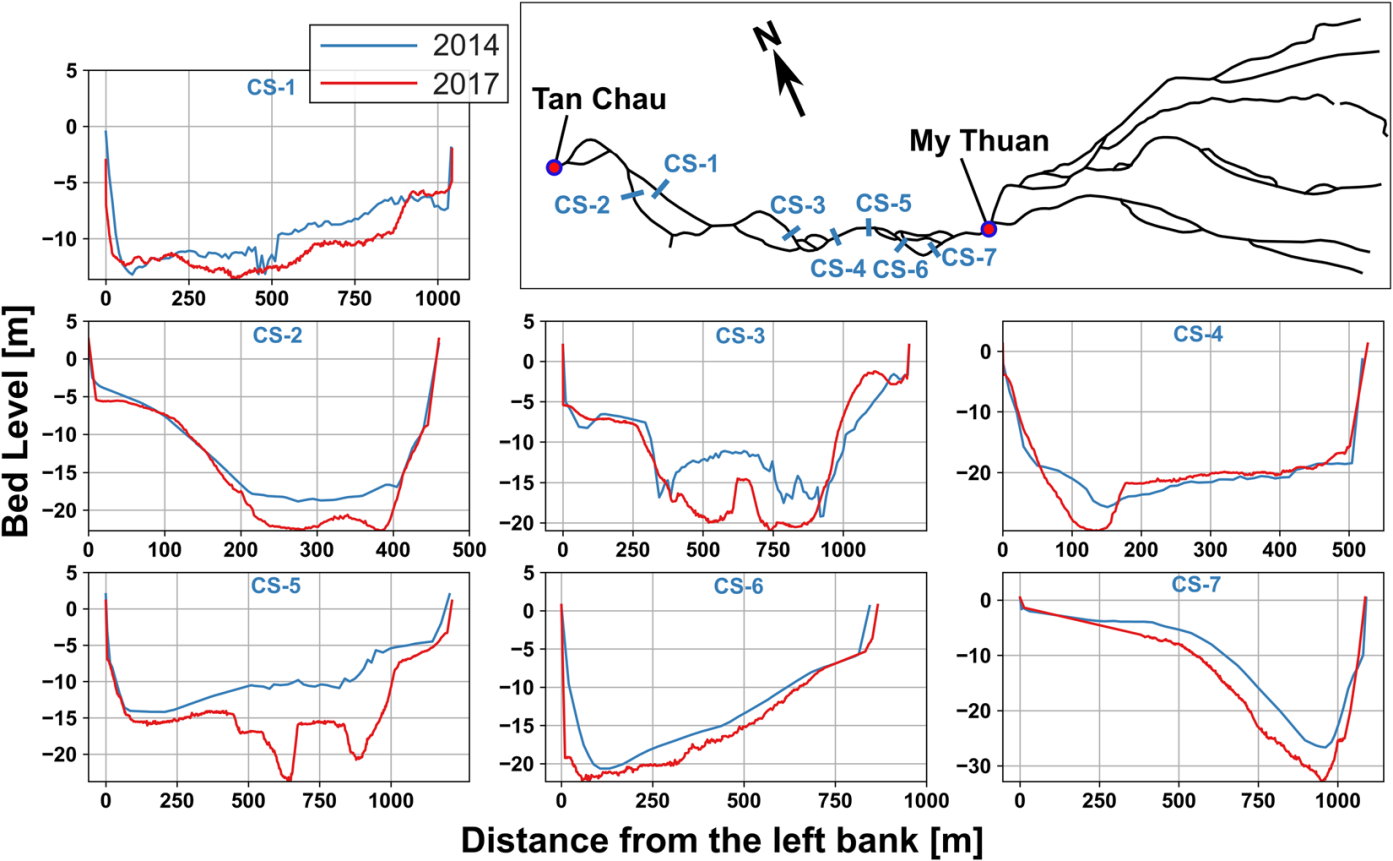
Cross-sections measured in 2014 versus 2017, showing channel deepening at various locations along the Tien River.

While morphological changes take place in complicated patterns of bed and bank erosion, a crude image of these changes can be inferred from developments of tidal signal. Vertical and horizontal tidal amplification at My Thuan and Tan Chau with increased tidal propagation speed along the Tien River and its estuarine distributaries imply that bed levels of the Tien River within the VMD and upstream in Cambodia have deepened significantly. This was also shown in previous research^7^ by comparing 1998 and 2008 bathymetries. The new evidence underscores continuity of the previously identified trends with even higher paces. From the tidal variation changes it can be concluded that the main changes between 1998–2008 occurred between 2004 and 2008. As further evidence for the extreme trends, Fig. 4 shows changes in several cross-sections, measured during 2014 and 2017 surveys (see methods). The cross-sections are measured at multiple pools along the meandering river, showing large incisions that may exceed average bed level changes along the river. Nevertheless, this complements the hypothesis of rapid and significant bed level changes within the VMD. Within the Hau River system, vertical tide has increased at Chau Doc but not at Can Tho. Tidal propagation speed has increased upstream of Can Tho and tidal discharge amplitude has increased at both Can Tho and Chau Doc. Therefore, we conclude that channel bed levels have deepened substantially upstream of Can Tho but not significantly downstream of Can Tho, which is also in-line with recent bathymetric surveys^51^ that find limited sources of modern sediment in lower Hau River. Hereafter, we show that the combined effect of deeper channels and amplified tides leads to increased SWI.

### Modelling tidal amplification and SWI

In absence of a complete recent morphological survey of the VMD, we used a calibrated state of the art barotropic model^52^ with 2006–2008 geometry (see methods) to physically replicate the observed tidal variations in response to bed level changes. The resulting hydrodynamic forces of the model were then used as boundary condition to an analytical SWI model^47^. In a number of geometrically schematised numerical experiments, the estuarine tidal response to bed level incision was quantified. To approximate the observed tidal amplifications, e.g., 20% and 50% increase of M_2_ tidal amplitude at My Thuan and Tan Chau (between 2005 and 2016) and 22 minutes’ reduction of tidal travel time between My Thuan and Tan Chau (Ch1 in Fig. 3), average bed levels must be lowered by up to 3 m (also see Methods and Supplementary Information). While in reality, morphological changes take place in sophisticated forms, with irregular combinations of bed and bank erosions, this exercise provides further evidence on how the Mekong tidal system adapts to bed level incision. Furthermore, due to complications of tidal dynamics in the multi-channel estuarine system, amplitude of tidal velocity changes asymmetrically among distributary channels. Using previous observations^53^ and the updated tidal forcing in the analytical model^47^, we demonstrate the combined impact of bed level change and tidal amplification on SWI.

**Figure 5:**
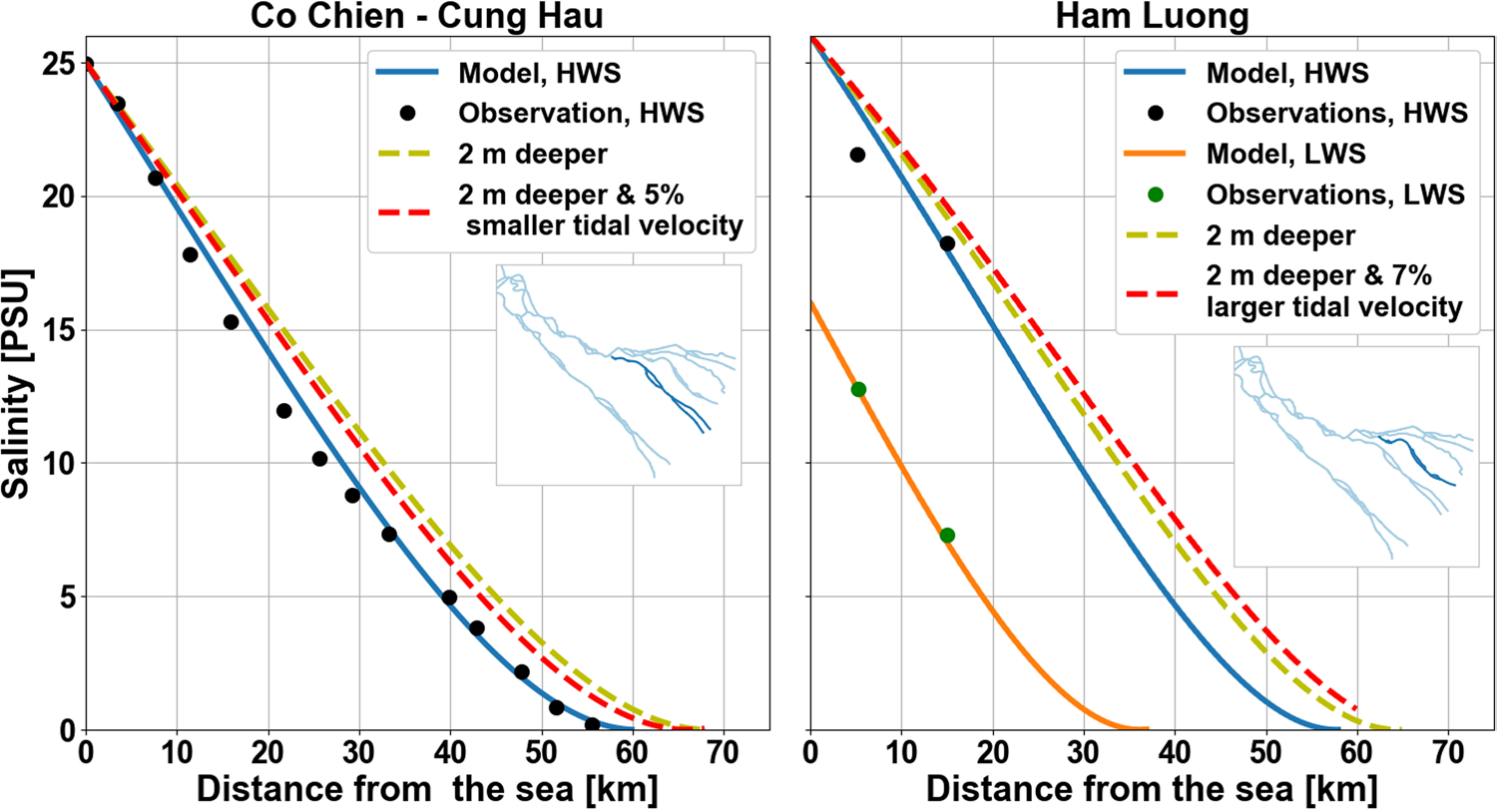
Observed^53^ SWI, during High Water Slack (HWS) and when available Low Water Slack (LWS), modelled longitudinal salinity profile in two different distributary channels of the Tien River in April 2005, compared to the expected longitudinal salinity profiles, given the new bed levels and the corresponding hydrodynamic response.

Two SWI longitudinal profiles^53^ during high water slack (HWS), from 2005, were reproduced using the analytical model (see Fig. 5). From the barotropic model we calculated that a 2 m deeper Co Chien-Cung Hau channel experiences 5% smaller tidal velocity (see Supplementary Information), leading to 5 km further SWI and c. 1.5 PSU higher salinity at the measurement station (about 20 km from the estuary mouth) during high water slack. Similar analysis in the 20% deeper Ham Luong channel results in 7% larger tidal velocity, hence, c. 10 km longer SWI and 2.5 PSU higher salinity 20 km from the sea. These estimates are close to the observed SWI trends of Fig. 1 (from 2005 to 2016) and systematically demonstrate how SWI responds to the combined effects of bed level changes and tidal amplification (also see Table 1). Note that Ham Luong has higher SWI increase rates than Co Chien-Cung Hau. The barotropic modelling shows that the two channels react differently to increasing depth. While normally tidal velocity amplitude decreases due to increasing depth, in Ham Luong, due to interaction with adjacent estuarine channels, tidal velocity amplitude increases. As larger depth increases SWI, the depth effect is moderated by tidal velocity amplitude drop in Co Chien-Cung Hau but magnified in Ham Luong.

**Table 1:** Summary of systemic decadal changes in different estuarine distributaries leading to SWI.

Co Chien-Cung Hau	Depth + 2 m↑ ∴ Tidal Vel. (5%)↓ ∴ SWI (5 km, 1.5 PSU at obs. station) ↑
Ham Luong	Depth + 2 m↑ ∴ Tidal Vel. (7%)↑ ∴ SWI (10 km, 2.5 PSU at obs. station) ↑

### Sediment starvation

Fluvial sediment supply to world deltas has reduced by 30%^54^. Upstream impoundments and downstream interventions (e.g., channel fixing with levees, diking the populated areas, sand mining) suppress delta aggradation rates^54–58^. Sediment dynamics of the MB are enigmatic due to data deprivation^25,36,59^ and its contrasting trends. The effects of sediment trapping by the dams under foreseeable future scenarios are expected to have already reached or reach the VMD within the next 10–20 years^60^. Flow regulations, e.g., modified flood pulse^15^ and shift of tropical cyclone tracks^27^ further reduce fluvial sediment supply. However, there are also processes that can still supply sediment to the VMD. Upstream erosion in response to sediment trapping, bank erosion due to climate variability^9^ (ENSO cold phases and enhanced intensity of monsoon) and sand mining^10^ and short term effect of mountain road constructions^61^ can delay downstream consequences, although not clear to what extent.

The earlier estimates^28^ of total sediment transported by MR were c. 160 Mt yr^−1^, but recent estimates^27,36,62,63^ range between 40–110 Mt yr^−1^. While only 13.5% of total VMD discharge springs from upper Mekong basin (Chinese Lancang-Mekong River), 40–60% of total Mekong sediment load originates from that stretch of the basin^5,60^. Estimates of sediment reduction rate from Chinese catchments due to 6 mainstream dams range between 40–90%^5,36,64,65^. Considering the hydropower development in Mekong tributaries, the aggregate sediment trapping of the dams are predicted to reduce sediment supply by 36%(moderate)−95%(worst case)^66^ and a recent study^67^ measured 75% reduction in sediment supply to the VMD. Furthermore, although sand export was banned in Cambodia and Vietnam, domestic consumption persisted. Projections to 2040^68^ show more than 1500 Mm^3^ demand within the VMD for infrastructure development. Figure 1 shows the spatial distribution of sand mining within the VMD and upstream in Cambodia. The issued mining licences in Vietnam, in 2015, summed up to 28 Mm^3^ yr^−1^ (40–50 Mt yr^−1^), which is four times the previous estimates^10^. Considering that this does not account for sand mining in Cambodia, Laos and possible illegal sand mining, only in Vietnam, this amount is likely to be close to 100% (or more) of the total fluvial sediment supply.

## Discussion

Examples of tidal amplification due to anthropogenic bed level incision, influencing SWI and tidal dynamics are seen, amongst others, in Ems, Elbe, Loire and Schelde estuaries^26^. We have related the observed increasing trends of SWI and tidal amplitudes to anthropogenic bed level degradation in the VMD as it was also envisioned in previous studies^7^. While this study does not quantify the contribution from sources of sediment starvation, we cannot ignore the concordance between bed level changes and the spatial distribution of sheer magnitudes of sand mining (Fig. 1) within the VMD. Although, temporal development of the observed trends also coincides with completion of major mainstream Lancang-Mekong dams, it is difficult to assume an immediate morphological response of the VMD to upstream dam construction. However, the combined effect of upstream sediment trapping, and downstream sand mining can lead to large scale channel erosions as it has also been shown for coastal erosion in various cases^58^.

**Figure 6:**
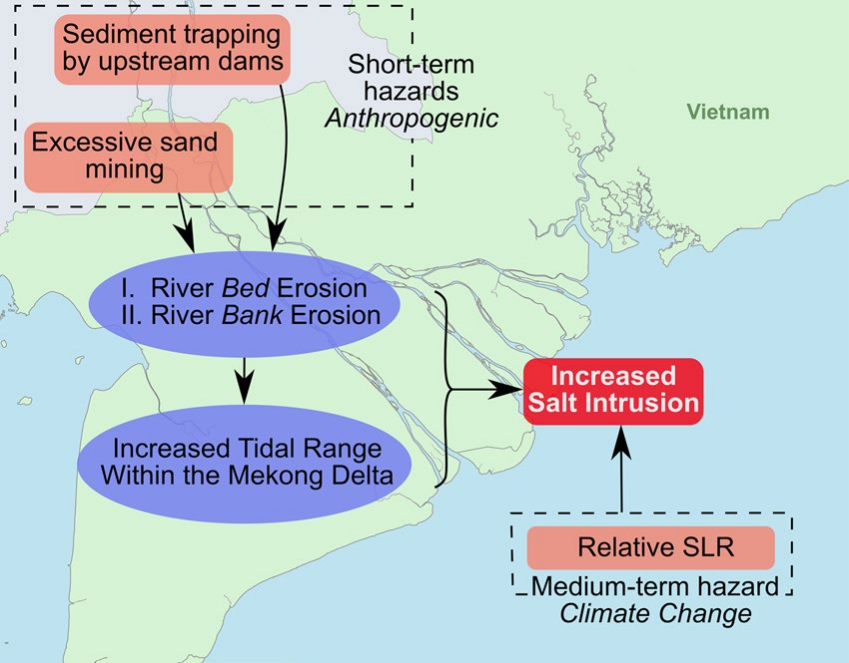
A summarizing diagram on the effect of various processes on SWI within the VMD.

While sea level is rising at nearly 3 mm yr^−1^^69^ and consequently, M_2_ tidal amplitude over the Mekong continental shelf is increasing by 1.2–2 mm yr^−1^^49^, the M_2_ tidal amplification within the delta is c. 2 cm yr^−1^ (e.g., at My Thuan), and salinity is rapidly increasing in the delta by 0.2–0.5 PSU yr^−1^, notwithstanding the increased upstream discharge due to CC and hydropower operations. The way SLR affects salt intrusion is a) by increasing channel depths, b) increasing offshore tidal amplitudes and c) increasing tidal amplitudes within the estuarine channels. Furthermore, the anthropogenic land subsidence^11,12^ possibly also contributes to deeper channels by c. 1–4 cm yr^−1^. On the other hand, in physical terms, sediment deficit has similar effects as relative SLR; i.e., it results in deeper channels and larger tidal amplitudes which lead to increased SWI. However, relative SLR (including land subsidence) in the past 15 years would perhaps sum up to c. 20–30 cm, while our presented evidence in this study, backed by previous research, indicates bed level incisions in the order of 2–3 m. Therefore, it is inevitable to conclude that anthropogenic trends outpace climate change trends by an order of magnitude and pose larger threats in the short term while exacerbating the medium to long term climate change impacts. Figure 6 provides a summary of various forces acting and influencing SWI within the VMD. While the effect of upstream developments is reaching Cambodia and the delta, sand mining seems to simultaneously jeopardize livelihood of the delta, calling for bold, multilateral and basin-wide management decisions before the consequences are irreversible or unadaptable.

## Methods

### Approach

We collected and depurated a large dataset of water level, discharge and salinity for detailed analysis. First, we assessed possible trends of highest measured salinity at various locations along estuarine network. Second, we extracted the temporal variation of discharge and tidal amplitudes (vertical and horizontal) at multiple stations as well as the tidal propagation speed along different channels. Next, we used a calibrated barotropic model^52^ of the VMD to relate tidal amplification to bed level changes. Through a number of quasi-synthetic sensitivity analysis simulations, we studied the barotropic response of the estuarine system to various scenarios of bed level changes. Eventually, we examined the effect of the barotropic changes on SWI using a widely-used analytical SWI model^47^. By looking at upstream sediment trapping and downstream sand mining distribution and recent morphological surveys in Tien and Hau Rivers, we related the existing trends to anthropogenic forces ahead of climate change impacts.

### Material

Water level, discharge and salinity measurement data within the VMD were originally collected by the Southern Regional Hydrometeorological Centre (SRHMC), Ho Chi Minh City, part of the Ministry of Natural Resources and Environment (MONRE), Vietnam. The data was provided by Southern Institute for Water Resources Planning (SIWRP), Ho Chi minh City, the advisory institute for the Ministry of Agriculture and Rural Development (MARD), Vietnam. The water level data within the VMD were collected in Hondau datum (Vietnamese benchmark system). The discharge data were obtained by translation of stationary water level and velocity data to cross-sectional discharge through rating curves. The rating curves were updated every quarter based on multiple days of continuous ADCP transect measurements. Discharge data of Kratie were equally generated from rating curves and originally provided by the Mekong River Commission (MRC). The stationary salinity data are 2-hourly manual over-depth measurement. The measured salinity profiles are then averaged as 1 × (salinity at 20% depth) + 2 × (salinity at 50% depth) + 1 × (salinity at 80% depth). Sand mining figures in Cambodia were extracted with permission from previous publications^10^. Sand mining figures within the VMD were reproduced from licenses issued in 2015 by MONRE and were analysed by SIWRP. The bathymetry data used for numerical barotropic modelling and analytical baroclinic modelling were provided by SIWRP. This dataset contains 1340 channels and 4308 cross-sectional profiles, mainly from 2006–2008, updated with more recent data when available^52^.

### Time series analysis

The peak of SWI within the VMD takes place in March and April of each year, when freshwater contribution to the VMD from the Tonle Sap Lake diminishes^2^, freshwater inflow is at its minimum and tidal amplitude is largest. Therefore, all time-series analyses were carried out for the period from beginning of March to end of April. Various parameters presented in the article were calculated as it reads below:

#### Cumulative discharge

Within the VMD, the hourly discharge signal was first de-tided using the low-pass Godin-filter^70^. Subtidal discharge was then integrated over the period of interest. This was done to minimize inaccuracies in rating curves, translating water level and point velocity to cross-sectional discharge. At Kratie, discharge was non-tidal and the daily value (07:00 AM every day) was simply integrated over a day.

#### Harmonic analysis

Tidal constituents were extracted from the tidal signal by least-square fitting of a tidal function, formed by leading independent harmonic constituents (satisfying Rayleigh criterion)^71^ to observed de-trended hourly tidal time series of water level or discharge. To isolate tidal amplitude changes within the estuary from those associated with marine system (e.g. SLR), for stations along Hau River, Can Tho and Chau Doc, tidal amplitudes are divided by tidal amplitude at the My Thanh offshore location (estuary mouth). For stations along the Tien River (Tan Chau and My Thuan), Binh dai station is considered as offshore station.

#### Trend analysis

To quantify the significance of trends, we performed a linear regression on the values using a nonparametric Mann-Kendall test and a Theil-Sen estimator^72,73^. This estimator computes the median of all pairwise slopes of values in time. The method is robust, i.e., it is not sensitive to outliers and does not require normality of the residuals. It is suitable for salinity and tidal amplitude data that are influenced by various processes^73^ (e.g., upstream discharge). For salinity, we performed a trend analysis on the P95 of salinity, i.e., the concentration not exceeded by 95% of the observations. We note that, similar trends were observed for P90 and P50 of salinity observations.

### Bathymetric survey

As part of assessing temporal and spatial variation of morphological changes of the VMD, several cross-sections were measured, during the rising stage of the wet season of 2014 and 2017. These observations were coordinated by Kyoto University. The boat-based surveys were conducted by RD-Instruments Workhorse Rio Grande 600 KHz Acoustic Doppler Current Profiler (ADCP), coupled with pole-mounted Trimble GPS for positioning. ADCP depth measurements were corrected for water level using the network of water level measurements along the river, operated by SRHMC.

### Modelling

Studying tides (de-trended water level fluctuations) instead of absolute water levels, shields the analysis against inconsistency in data referencing (e.g., due to SLR or land subsidence) providing strong evidence to changing morphology. We used previously published^52^ detailed barotropic model of the VMD to examine sensitivity of the estuarine tidal system to bed level changes. For detailed model description, set-up, calibration and validation we refer to the underlying publications^52,74^. While morphological changes take place with spatial along and cross-channel variability, in absence of a recent bathymetry, we performed this exercise under idealized circumstances in order to show how the system responds to bed level changes. The choices for bed level changes were inspired by the linearized asymptotic solution for phase speed in a long, intermediate-depth, near-equilibrium estuary^50^. We divided the channels into multiple stretches (as tidal propagation time was calculated in Fig. 3). We subsequently calculated the required average bed level changes that result in the observed reduction in tidal travel time. The bed levels of each stretch were lowered step-wise and in turn in the barotropic model. The variation of horizontal and vertical tide as well as tidal travel time were then compared to the observations to arrive at a reasonable comparison to the observations (see supplementary information).

Since SWI trend analysis was carried out for P95, which approximately corresponds to high water slack, it was possible to apply the Savenije^47,53^ slack tide SWI theory to study the saline water intrusion physical processes. Savenije^47^ showed that the steady-state salt balance equation relates maximum SWI to horizontal salinity gradient and derived a predictive model for the varying along-channel dispersion coefficient. The dispersion coefficient is a function of upstream discharge, average depth, channel geometry and tidal excursion (a function of tidal velocity amplitude). The model^47^, (perhaps the most calibrated SWI model around the world), using 2005 measurements^53^, was calibrated for multiple distributaries of the Mekong estuarine system. To show the sensitivity of SWI, we updated the calibrated analytical model of 2005^53^ by feedback from the bed level changes and the barotropic response for tidal velocity amplitude (see Fig. 5). Since we aimed to look at the effect of bed level changes and the associated tidal variations, other boundary conditions were kept constant.

## Supplementary information

Supplementary Information

## Data Availability

The underlying gauge data (observed water level, discharge and salinity) provided by the SIWRP, following the organizational policy, can only be provided upon request for non-commercial use. DFlow-FM is an open source numerical model. Nevertheless, all codes, data and models can be provided to the reviewers for any validation or reproduction.
