# Supplementary material for: Tidal amplification and salt intrusion in the Mekong Delta driven by anthropogenic sediment starvation
Source: Sci Rep. 2019 Dec 10;9:18746. doi: 10.1038/s41598-019-55018-9 (PMC6904557; doi:10.1038/s41598-019-55018-9)
Supplement: Supplementary file 1 — Supplementary Information [file 41598_2019_55018_MOESM1_ESM.pdf]

Supplementary Information

Manuscript Title: Tidal amplification and salt intrusion in the Mekong Delta driven by anthropogenic sediment starvation

Authors: Sepehr Eslami*, Piet Hoekstra, Nam Nguyen Trung, Sameh Ahmed Kantoush, Doan Van Binh, Dung Do Doc, Tho Tran Quang and Maarten van der Vegt

Historical trends

To further illustrate upstream trends due to climate change and hydropower operation, and downstream trends driven by human intervention or SLR, we studied long term variation of upstream discharge and downstream water levels. Figure S1 shows upstream cumulative and minimum discharge at Kratie in the upper panel, hourly water level and dry season M_2 tidal amplitude at an offshore station (Binh Dai, see Figure 1 of the main article for location) and water level and dry season M_2 tidal amplitude at My Thuan and Can Tho in the two lower panels. Despite temporary reduction of freshwater supply upon completion of Jinghong and Xiaowan dams in 2010, dry season discharge at Kratie (cumulative and P05) shows a consistent increasing trend over the past three decades, accentuating the fact that freshwater supply does not explain observed SWI trends. Temporal variation of M_2 tidal amplitude at Binh Dai shows an increasing trend in the past two decades, aligned with published[1] tidal amplification trends in response to SLR (1.2-2 mm/yr). Since tidal amplitude generally descends upstream[2], it is expected that tidal amplitudes at Can Tho and My Thuan follow similar trends as those associated with SLR. While at Can Tho the general increase in M_2 tidal amplitude is in line with SLR trends, at My Thuan, there is a sudden break-away from the SLR trend from 2008 onwards. Combined with variations in tidal propagation speed and tidal discharge variations, using a numerical barotropic model we show that this sudden deviation is associated with anthropogenic bed level changes within the VMD.

Barotropic modelling

Figure S2 shows barotropic response of the model to bed level changes for one of the simulations that was close enough to the observations. Bed level changes influence the vertical and horizontal tide at four major stations (top left panel) and tidal velocity amplitude changes at the mouth of seven different estuarine channels (middle left panel). It also shows the reduction of tidal travel time along seven stretches as in Figure 3 of the main article. The exercise can demonstrate that although the exact tidal variations cannot be reproduced (when bathymetry is not fully updated), the general observed trends can be shown with simple lowering of bed levels. The relative tidal variation is maximum in Tan Chau and Chau Doc where tidal amplitudes are smaller, and at Can Tho, the relative vertical tide does not change. Note that while we do not try to reproduce perfect match between the model and observations, also the observed variation percentages, e.g., due to variations in freshwater inflow, are sensitive to the choice of reference and target years. For example, tidal discharge amplitude increase from 2005 to 2016 is ~40% while to 2015 is ~30%. Nevertheless, tidal travel times that are more dependent on average bed level changes show good comparison to the observations. While Ch1 (between My Thuan and Tan Chau) is least sensitive to bed level changes, tidal travel time along lower Tien River distributaries show larger response to bathymetric changes. The bed level variations are in the same order of magnitudes that was estimated using a first order linearized estimate based on tidal propagation speed. Nevertheless, within the multi-channel estuarine system, where tidal dynamics are more complicated due to estuarine channel interactions, a calibrated numerical model is often required to capture these dynamics as presented here.

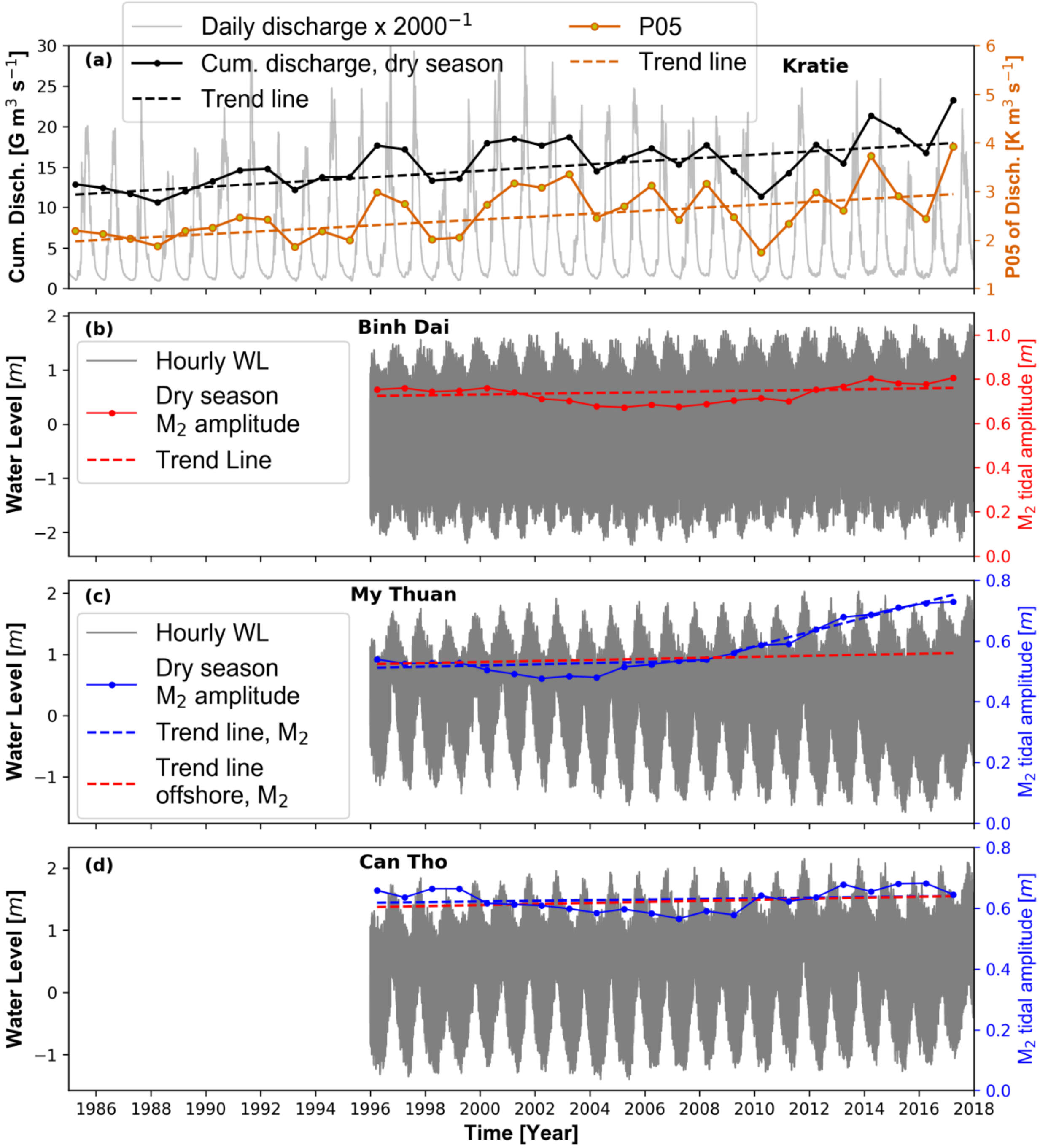

Figure S1, Variation of upstream freshwater supply and downstream tidal amplitudes; (a) Daily discharge and the dry season cumulative discharge in Kratie; (b) water level and the dry season M_2 amplitude of tidal signal at Binh Dai offshore station; (c) & (d) water level and dry season M_2 amplitude and their trends against expected SLR tidal amplification within the VMD at My Thuan (c) and Can Tho (d).

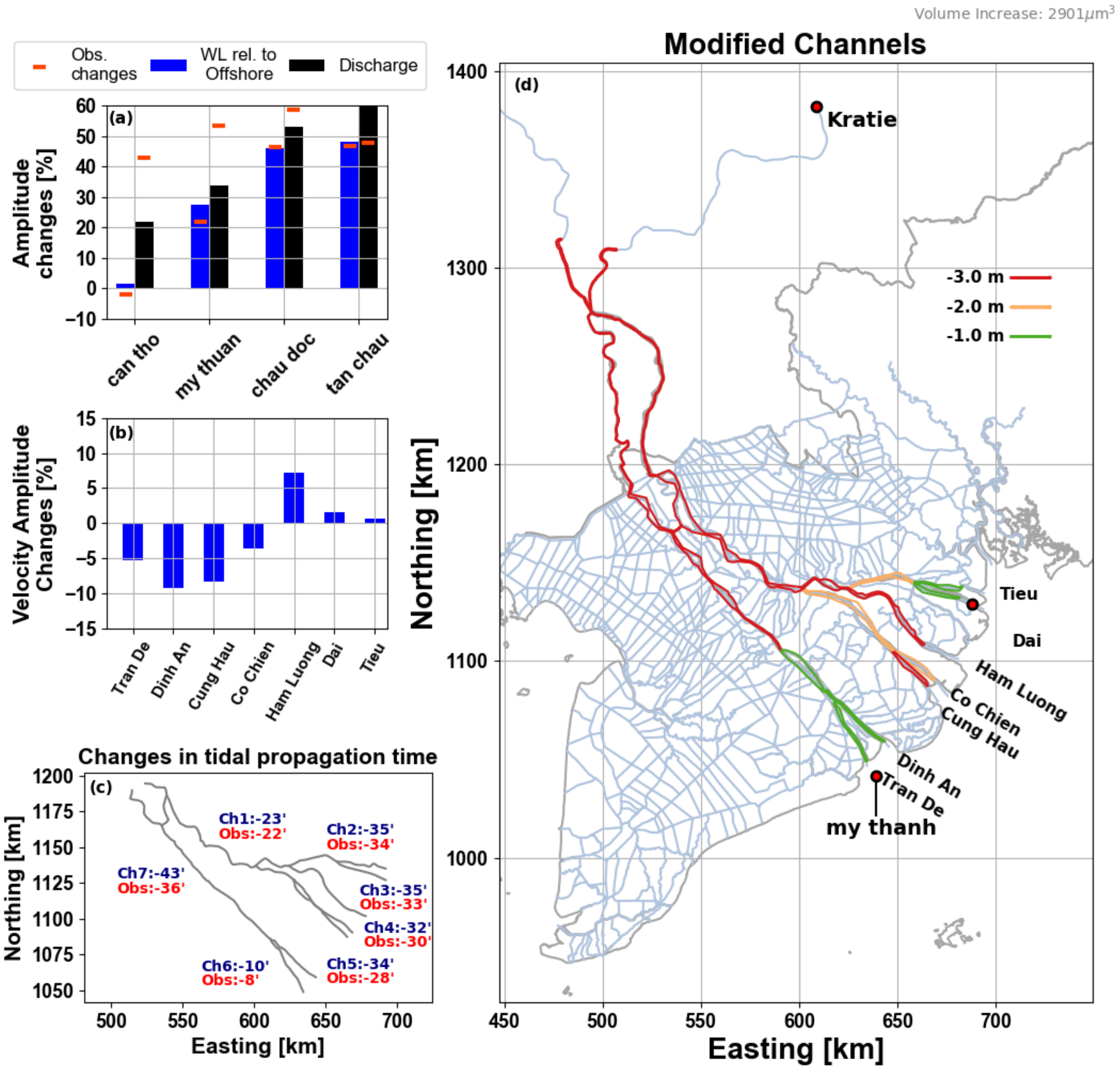

Figure S2, Barotropic model response to bed level changes; (a) variation in M_2 tidal water level amplitude relative to offshore and M_2 tidal discharge relative to the year 2005, compared to the observed changes between 2005 and 2016; (b) M_2 tidal velocity amplitude change at the mouth of various estuarine distributary channels in response to bed level changes; (c) calculated (blue) change (reduction) of tidal propagation time along different stretches (between consecutive stations) compared to the observed reduction (red) identical to the stretches of Figure 3 in the article; (d) applied bed level changes [in meters] as highlighted by different colours along different channels of the Mekong Delta estuarine system.

References

1. Nhan, N. H. Tidal regime deformation by sea level rise along the coast of the Mekong Delta. *Estuar. Coast. Shelf Sci.* **183**, 382–391 (2016).

2. Eslami, S. *et al.* Flow Division Dynamics in the Mekong Delta: Application of a 1D-2D Coupled Model. *Water* **11**, (2019).
